# Discrepancies between cortical and behavioural long‐term readouts of hyperalgesia in awake freely moving rats

**DOI:** 10.1002/ejp.892

**Published:** 2016-05-05

**Authors:** B. Ljungquist, T. Jensen, L. Etemadi, J. Thelin, G. Lind, M. Garwicz, P. Petersson, F. Tsanakalis, J. Schouenborg

**Affiliations:** ^1^ Neuronano Research CentreSection for NeurophysiologyLund UniversitySweden

## Abstract

**Background:**

It is still unclear to what extent the most common animal models of pain and analgesia, based on indirect measures such as nocifensive behaviours, provide valid measures of pain perception.

**Methods:**

To address this issue, we developed a novel animal model comprising a more direct readout via chronically (>1 month) implanted multichannel electrodes (MCE) in rat primary somatosensory cortex (S1; known to be involved in pain perception in humans) and compared this readout to commonly used behavioural pain‐related measures during development of hyperalgesia. A translational method to induce hyperalgesia, UVB irradiation of the skin, was used. Localized CO_2_ laser stimulation was made of twenty skin sites (20 stimulations/site/observation day) on the plantar hind paw, before and during the time period when enhanced pain perception is reported in humans after UVB irradiation.

**Results:**

We demonstrate a 2–10 fold significant enhancement of cortical activity evoked from both irradiated and adjacent skin and a time course that corresponds to previously reported enhancement of pain magnitude during development of primary and secondary hyperalgesia in humans. In contrast, withdrawal reflexes were only significantly potentiated from the irradiated skin area and this potentiation was significantly delayed as compared to activity in S1.

**Conclusions:**

The present findings provide direct evidence that chronic recordings in S1 in awake animals can offer a powerful, and much sought for, translational model of the perception of pain magnitude during hyperalgesia.

**What does this study add?:**

In a novel animal model, chronic recordings of nociceptive activity in primary somatosensory cortex (S1) in awake freely moving rats are compared to behavioural readouts during UVB‐induced hyperalgesia. Evoked activity in rat S1 replicates altered pain perception in humans during development of hyperalgesia, but withdrawal reflexes do not.

## 1 Introduction

Animal models of pain are extensively used to explore new drug targets and to evaluate new treatments of pain. However, the translation of pain research findings from animal models to humans has, over the past decades, often been disappointing and many candidate drugs that appeared to be active as analgesics in animals have turned out to be inefficient in humans (Woolf, 2010).

One factor potentially contributing to poor translatability may be that most current models of pain in awake animals used for development of pain treatments are based on behavioural measures of perceived pain, such as nociceptive withdrawal reflexes or other pain‐related behaviours (Kakigi et al., 2004; Sandrini et al., 2005). Although human nociceptive motor responses can be shown to correlate to perceived pain under laboratory conditions, it is still unclear to what extent motor responses provide a complete picture of perceptual aspects of pain (Weng and Schouenborg, 1998; Sandrini et al., 2005) across a wider range of natural conditions.

Consequently, there is a need to develop animal models allowing long‐term assessment of central nervous mechanisms underlying perceptual aspects of pain in the awake, freely moving animal. Such animal models would be highly useful for the analysis of how the presence of prolonged nociceptive stimulation may lead to altered pain perception, such as during the establishment of hyperalgesia and/or the development of chronic pain.

Therefore, the aim of this study was to characterize changes in nociceptive‐evoked activity in primary somatosensory cortex (S1) during hyperalgesia‐like conditions in awake, freely moving animals and to analyse how these changes compare to changes in conventional behavioural (withdrawal reflex) measures. The rational for choosing S1 as target of our investigation was that there is strong evidence from human studies that implicate S1 in perception of pain magnitude (Lenz et al., 1995; Apkarian et al., 2005; Nir et al., 2008). From animal studies it is known that this cortical area receives a somatotopically organized nociceptive input from Aδ (Handwerker and Zimmermann, 1972; Qiao et al., 2008; Shaw et al., 2001) and C fibres mediated by multiple ascending spinal pathways (Kalliomäki et al., 1993b; Schouenborg et al., 1986) and contains neurones that can encode stimulus intensity (Kuo et al., 2009; Zhang et al., 2011; Wang et al., 2003) .

To be able to monitor nociceptive input to S1 in the awake freely moving animal we used ultrathin, biocompatible multichannel electrodes that can be implanted in the nervous tissue for a long period of time (Lind et al., 2010). An established translational method to induce hyperalgesia, UVB irradiation, for which there are detailed characterizations both of the nociceptive responses in rat spinal cord, primary somatosensory cortex, most importantly, of the sensory changes in humans, was used (Bishop et al., 2007; Jensen et al., 2011; Gustorff et al., 2013; Sikandar et al., 2013; Van Den Broeke et al., 2015; O'Neill et al., 2015; Lopes and McMahon, 2016).

We report significant differences in time course and spatial distribution between nociceptive cortical activity and conventional behavioural measures of pain during UVB‐induced hyperalgesia in awake, freely moving animals. Importantly, alterations in nociceptive cortical activity, but not in reflex activity, were found to closely correspond to previously described alterations of pain perception in human studies after UVB irradiation (Gustorff et al., 2013).

## 2 Methods

### 2.1 Ethical approval and animals used

Ethical approval (registration number M12‐10), for the experiments was obtained in advance from the Lund/Malmoe local ethical committee on animal experiments, regulated by the Swedish Board of Agriculture (see Supporting Information Appendix S1). Seven female Sprague–Dawley rats (Taconic, Silkeborg, Denmark), weighing 230–280 g were used in this study. (See Supporting Information Appendix S1).

### 2.2 Indices of pain and pain‐related phenomena

**Figure 1:**
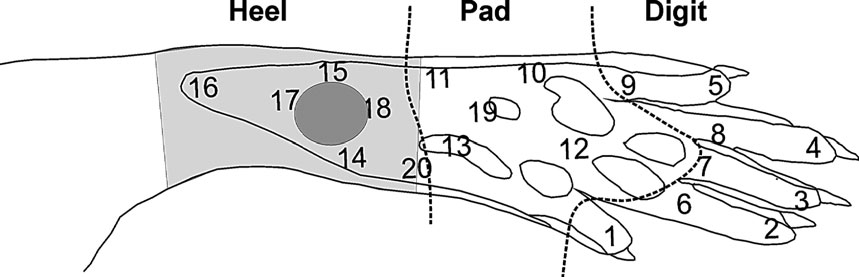
UVB irradiation and stimulation sites on the right hind paw of the rat. The UVB‐ irradiated area, around the heel, is shaded in light grey; the laser Doppler measurements were taken from this area. Numbers indicate stimulation sites for the CO_2_ laser; dashed lines divide the numbers into three areas of the paw – Heel, Pad and Digit. The ellipse in dark grey indicates where mechanical and thermal stimulation in the behavioural tests was performed.

The experimental protocol was devised so as to allow a quantitative characterization and comparison of how UVB irradiation of the proximal part of the hind paw (Fig. 1, see also Supporting Information Appendix S1), at an intensity causing an inflammatory reaction but no blister, affects different indices of pain and pain‐related phenomena. The two main indices compared were behavioural tests (see below) and local field potentials and neuronal spikes in S1 evoked upon mechanical and noxious stimulation of the skin. Local blood flow was assessed to determine the degree of inflammation in the skin after UVB irradiation (see Supporting Information Appendix S1).

Behavioural tests were performed before surgery for electrode implantation in S1, after surgery and on days 1, 2, 4 and 7 after UVB irradiation. Baseline electrophysiological recordings were performed twice before UVB irradiation, separated by 1–2 days and on days 1, 2, 4 and 7 after irradiation. Local blood flow was assessed before UVB irradiation and on day 1 after irradiation.

### 2.3 Behavioural tests

To measure withdrawal latency to radiant heat and mechanical thresholds the Hargreaves plantar test (Hargreaves acrylic cubicle, plantar test cat. 7370) and the dynamic plantar aesthesiometer (Ugo Basile, Monvalle, Italy) were used. Rats were placed in clear cubicle and left to acclimatize for 10 min or until they had stopped to explore their environment. Stimuli were delivered only when the animals showed no voluntary motor activity. For more details see Supporting Information Appendix S1.

#### 2.3.1 Blood flow measurements

To obtain a measure of the degree of inflammation in the skin, blood flow was measured using a laser Doppler flow meter (Moors‐LDF, Moor instruments, Axminster, UK) in anaesthetized (1.1–1.5% isoflurane in a mixture of 40% oxygen and 60% nitrous oxide) rats. A cutaneous probe (VP1T/7) was used to record 10 s each from three different sites on the right heel. An average flux was calculated from these data.

### 2.4 Electrophysiological recordings

Electrophysiological recordings of local field potentials and neuronal spikes in S1 were conducted in freely moving rats in a cubicle (19 × 20 × 14 cm) with an open top. During the test trial, rats could move around in the chamber while tethered to the recording wire. Stimuli were delivered only when the animal showed no voluntary motor activity, therefore the interstimulus intervals were ≥1 s. Each of 20 sites (Fig. 1) on the glabrous skin of right hind paw were stimulated 20 times with a CO_2_ laser.

#### 2.4.1 Assessment of nociceptive input to S1 using CO_2_ laser stimulation

A CO_2_ laser (see Supporting Information Appendix S1) with a 10 W output power was used to elicit nociceptive neural activity and C fibre‐evoked potentials. This method allows a precise temporal and spatial stimulation, selective for cutaneous nociceptive Aδ and C afferent fibres (Schouenborg et al., 1992). To obtain a high degree of compatibility with other studies in the field, we used standard reflex threshold tests that are widely used to assess pain in awake animals. The threshold was defined as the pulse duration of CO_2_ laser stimulation that elicited a withdrawal response in three out of five trials in each animal (usually 26–30 ms). These stimulation energies are well tolerated by the awake animals and have been shown to evoke late cortical field potentials in the rat S1 through the activation of cutaneous nociceptive C fibres in the normal (Kalliomäki et al., 1993a,b) and in the hyperalgesic state (Jensen et al., 2011). Strong stimulation intensities were avoided to not cause unnecessary animal suffering. All elicited withdrawal reflexes were noted.

### 2.5 Electrodes and implantation

A microwire array electrode was built in house. The electrode consisted of 29, 12 μm platinum‐irridium wires insulated with parylene C (Paratech, Järfälla, Sweden) and embedded in gelatine type A (2%; Sigma‐Aldrich Co, Saint Louis, MO, USA) for optimal stiffness during insertion into the cortex (Lind et al., 2010). For detailed procedures see Supporting Information Appendix S1.

Seven rats were mounted in a stereotactic frame and implanted with a microwire array electrode in S1. These animals were anaesthetized i.p. with 6.3 mL/kg solution of 1 mg/mL Domitor vet (medetomidin hydrochloride; Orion pharma, Turku, Finland) and 50 mg/mL fentanyl (Braun, Aschaffenburg, Germany). For detailed surgical procedures and postsurgical care see Supporting Information Appendix S1.

### 2.6 Data and statistical analysis

Analysis of behavioural tests was carried out using GraphPad Prism 5.04 (GraphPad Software, San Diego, CA, USA) and results are expressed as means ± standard error of the mean (SEM). One‐way anova followed by a Tukey′s post hoc test was used for statistical analysis of behavioural tests (*p* < 0.05 was taken as significant, 95% confidence interval; *n* = 7 for both groups). Paired student t‐test was used for statistical analysis of blood flow measurements (*n* = 6, as data from one time point was not collected). The frequency of withdrawal responses elicited by CO_2_ laser stimulation evoked during S1 recordings were analysed with one‐way anova followed by Bonferroni′s multiple comparison test. Numbers of reflexes were expressed as percentages of the total number of stimulations in each area.

### 2.7 Cortical recordings

Data trials from three animals were rejected as they showed large contaminating artefacts due to abiotic failures. Therefore, local field potentials (LFPs) and neuronal spikes from four animals were included in the analysis. LFPs were acquired using a 28‐channel microelectrode and a Neuralynx Digital Lynx 10S acquisition system. For details on sampling, filtering and rejection criteria see Supporting Information Appendix S1.

Electrophysiological data were stored together with experimental meta‐data, such as animal data and recording conditions, in a MySQL database for facilitation of grouped analysis. Averaged evoked field potential responses were calculated using in‐house developed software written in MATLAB (Mathworks, Natick, MA, USA). Response magnitude was calculated as area under curve (AUC) using onset and end, and using the onset potential value as baseline. If the potential did not return to baseline after onset, the end of the response was set to 500 ms. For definitions of onset and offset see Supporting Information Appendix S1.

A two‐sample Mann–Whitney *U*‐test with correction for multiple comparisons (*n* = 4) using Bonferroni correction was used to evaluate if the chosen parameters were significantly different from values before UV exposure, yielding separate *p*‐values for each animal. To combine the yielded *p*‐values, Stouffers method for meta‐analysis of *p*‐values (Stouffer et al., 1949) was performed for combining evidence from separate sources (animals). This method was necessary since the amplitude of the potentials showed large inter‐individual variance.

### 2.8 Spike population and single unit activity analysis

In addition to the field potentials, spikes sampled at 32556 Hz were recorded with the same acquisition system. The spikes were extracted through threshold detection. Threshold were set automatically as single sided thresholds at −4SD using the median based SD estimation method (Quiroga et al., 2004). This data was stored together with the LFP data in the previously mentioned database, and also imported to and analysed using MATLAB.

The spike data were then imported from the recording database to a MATLAB data structure using the Database toolbox. After import, the channel spike trains were automatically spike sorted using the k‐means based approach supplied by the Fieldtrip toolbox (Oostenveld et al., 2011). Units were discarded if the ratio of inter‐spike intervals <1.5 ms of the unit exceeded 0.5%.

Peristimulus time histograms for the identified units were then calculated using 10 ms bins. To identify the unit as a unit responding to either Aδ‐ or C‐fibre input, the bins corresponding to the time for the respective input were summed up as follows. Aδ‐response was defined as having a significant response compared to baseline during bins from 10 to 50 ms and C‐fibre during bins from 200 to 600 ms. The baseline response was summed up using an interval of bins from 380 to 50 ms before the stimulus. Significance for response to stimuli was analysed using a two‐sample Kolmogorov–Smirnov test using a significance level of 0.05.

The summed response across animals of the significantly responding units were then grouped according to stimulation site, fibre type and days after UV radiation exposure. For the units which then were significantly responding to nociceptive input the response before and after UV stimulation was analysed. In similarity to the previous analysis for evoked potentials, a two‐sample *t*‐test, after taking the logarithm of the data, with correction for multiple comparisons (*n* = 4) using Bonferroni correction was used to evaluate if the response for the grouped units were significantly different from values before UV exposure.

## 3 Results

Nociceptive activity in primary somatosensory cortex (S1) evoked by CO_2_ laser stimulation of the skin was recorded before and during UVB‐induced hyperalgesia in awake, freely moving rats using chronically implanted electrodes in cortical lamina V. The reason for choosing lamina V for recordings was that nociceptive neurones have been reported to be concentrated in this lamina in rats (Lamour et al., 1983a,b; Moxon et al., 2008). Nociceptive C fibre evoked multiunit activity and local field potentials similar to those previously found in anaesthetized rats (Jensen et al., 2011; Kalliomäki et al., 1993a,b), with respect to latency and duration, were observed in the awake animals after CO_2_ laser stimulation during control conditions. As in anaesthetized rats, early but irregular potentials and neural activity, presumably caused by nociceptive Aδ fibres, were also evoked after CO_2_ laser stimulation. These early responses were not analysed further here.

### 3.1 Altered spike unit activity and local field potentials in S1 after UVB irradiation of the skin

The skin of the heel of the hind paw was irradiated with UVB in all seven rats. Twenty different sites on the hind paw (Fig. 1) were stimulated with a CO_2_ laser to evoke spike unit activity and local field potentials in S1. The different sites on the paw were stimulated before and on a series of days after UVB irradiation in a semi random order. Each site was stimulated in total 20 times per recording day. Spike unit activity and local field potentials elicited in the hind paw region of S1 upon skin stimulation were analysed separately for the heel, pad and digit areas of the paw (Fig. 1). These three areas represent skin areas where primary hyperalgesia (Heel) and secondary hyperalgesia (Pad and Digits) may be expected to occur. Electrophysiological data from three animals were rejected as they showed large contaminating artefacts due to technical failures and were hence not included in the results.

**Figure 2:**
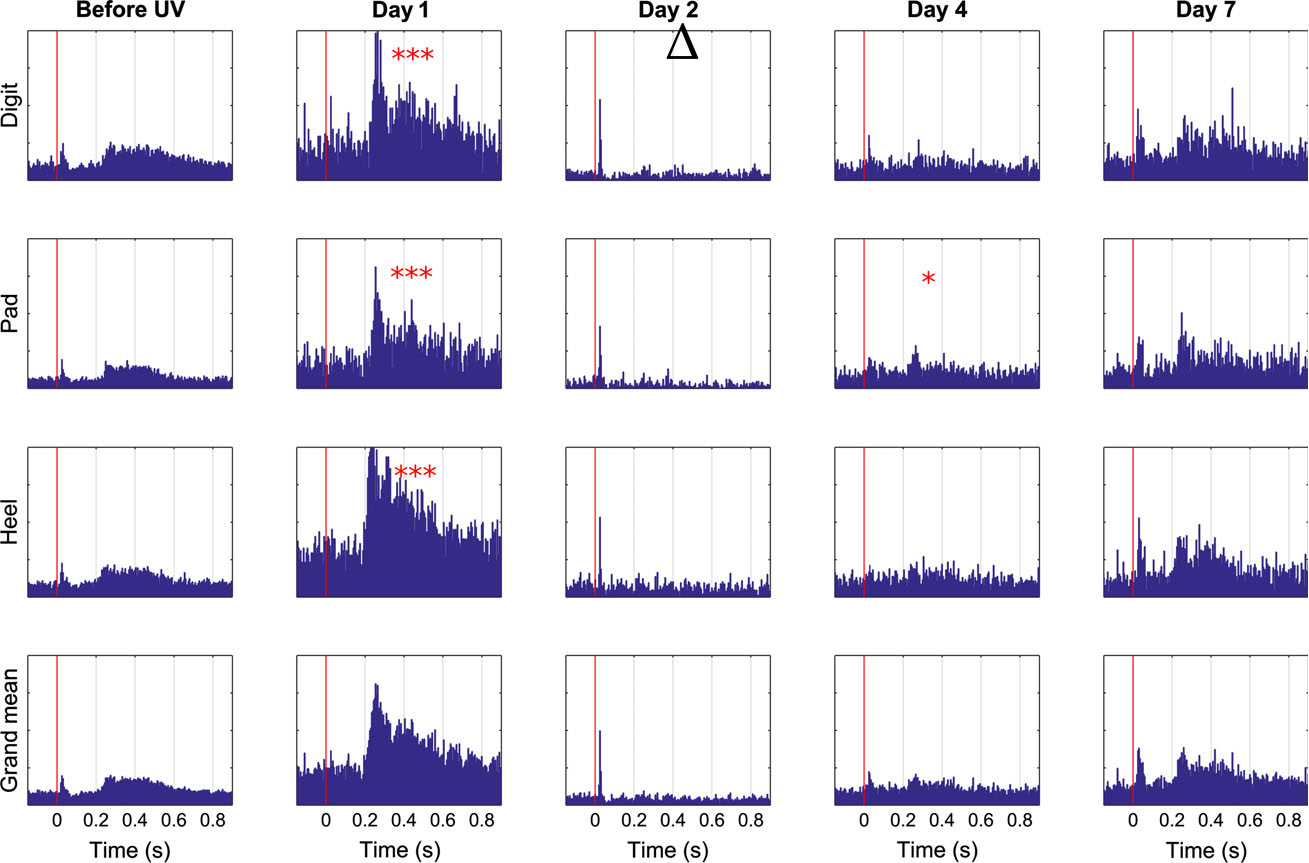
Mean time course of changes in unit activity of neurons in SI cortex upon CO_2_ laser stimulation following UVB irradiation of the hind paw skin. Time progression from left to right; panels to the left represent control conditions before UVB irradiation (but after surgery and implantation, cf. Figs. 4‐6), dashed line indicates UVB irradiation, panels to the right of dashed line depict consecutive test occasions over a period of 1 week postirradiation (Days indicated on top). Peristimulus time histograms upon neuronal responses to stimulation of sites 1–9 (Digit cf. Fig. 1), sites 10–13 and 19 (Pad), sites 14–18 and 20 (Heel). For each of the 20 sites 20 stimulations were given per day and animal. The data from all sites belonging to a given area was pooled. Bottom row shows a grand mean of all three groups. *X*‐axis in individual diagrams denotes time in seconds and the *y*‐axis depicts amplitude (μV). Red vertical line indicates time of stimulation. *p < 0.05, ***p < 0.001significant increase and ▵ <0.05 significant decrease.

Before UVB radiation, in control conditions, a modest but distinct response of spike units was seen upon C fibre stimulation of any of the three areas of the paw – Digits, Pad and Heel (Fig. 2). Onset latencies of these responses were about 200 milliseconds on Heel stimulation and somewhat longer from the other two areas. Responses lasted around 400 milliseconds. On Day 1 following UVB irradiation, evoked spike unit activity from the primary area (Heel) was increased by a factor of approximately 10 and slightly less from the secondary areas (Digits and Pads) as compared to control, with a significance of *p* < 0.001 (entry a) in Supporting Information Table S1) for all areas. Also baseline activity was significantly (*p* < 0.05, entry j) in Supporting Information Table S1) increased during stimulation of Heel. There were no statistically significant increases in spike unit activity from any area on any of the remaining days investigated except Day 4, Pad (*p* < 0.05, entry a) in Supporting Information Table S1). Already on Day 2 the evoked unit activity was back to control levels, or as in the case of responses elicited from digits, significantly (*p* < 0.05, entry i) in Supporting Information Table S1) below control level. On this day, also baseline activity before stimulation was lower as compared to before UVB exposure.

**Figure 3:**
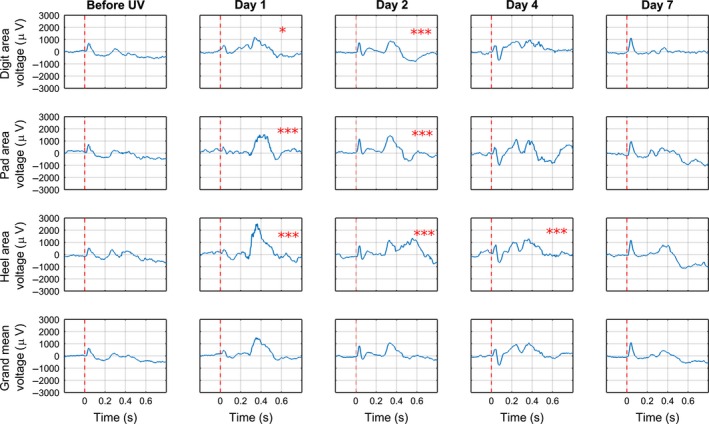
Mean time course of changes in evoked potentials evoked in SI cortex upon CO_2_ laser stimulation following UVB irradiation of the hind paw skin. Time progression from left to right; panels to the left represent control conditions before UVB irradiation (but after surgery and implantation, cf. Figs. 4‐6), dashed line indicates UVB irradiation, panels to the right of dashed line depict consecutive test occasions over a period of 1 week postirradiation (Days indicated on top). For each of the 20 sites 20 stimulations were given per day and animal. The data from all sites belonging to a given area sites 1–9 (Digit cf. Fig. 1), sites 10–13 and 19 (Pad), sites 14–18 and 20 (Heel) and day were averaged. Bottom row shows an average of all three groups. *X*‐axis in individual diagrams denotes time in seconds and the *y*‐axis depicts amplitude (number of spikes). Red vertical line indicates time of stimulation. *p < 0.05, ***p < 0.001 significant increase.

The local cortical field potentials evoked upon C fibre stimulation from any of the three areas of the hind paw were rather small in control conditions, before UVB irradiation (Fig. 3). In contrast, on Day 1 following UVB irradiation, distinct local field potentials were evoked from all three areas, and were seen as large positive deflections with an onset latency of about 260 milliseconds (from Heel and somewhat longer from Pad and Digits) and duration of about 300 milliseconds. The area under the curve (AUC) of the response from Heel was increased by a factor of more than five on Day 1 as compared to control, whereas responses from Pad and Digits on this day were increased by a factor of more than three and two, respectively, as compared to control (*p* < 0.001 for pad and heel, *p* < 0.05 for digits, entry b) in Supporting Information Table S1). On Day 2, responses evoked from the heel were clearly smaller than on Day 1, whereas on approximately the same level for pad and digits, but still significantly increased (*p* < 0.001 for all areas, entry b) in Supporting Information Table S1) compared to control. On Day 4, only responses evoked from Heel were significantly increased (*p* < 0.001, entry b) in Supporting Information Table S1) as compared to control, but only by a factor of 1.5. On Day 7 none of the local cortical field potentials upon C fibre stimulation were significantly different from control conditions.

### 3.2 Altered nocifensive responses to mechanical and thermal nociceptive stimulation after UVB irradiation of the skin

**Figure 4:**
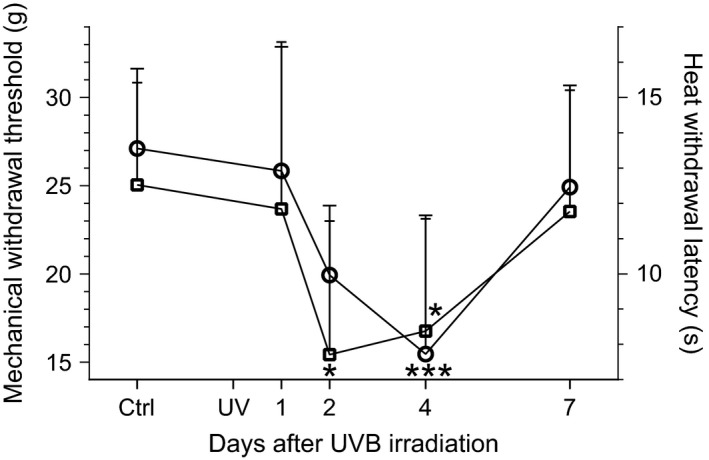
Time course of changes in behavioural nociceptive responses following UVB irradiation of the hind paw skin. Mean and SEM of withdrawal thresholds to mechanical stimulation (circles; left *y*‐axis) and withdrawal latencies to heat stimulation (squares; right *y*‐axis) are plotted; time scale after UVB irradiation is linear. The control (Ctrl) value before UVB irradiation (UV) represents a mean of 11 test occasions performed postsurgery (cf. Supporting Information Fig. S1). *p < 0.05, ***p < 0.001; one‐way anova (entry d) resp. e) in Supporting Information Table S1) followed by a Tukey′s post hoc test; *n* = 7 for both groups.

**Figure 5:**
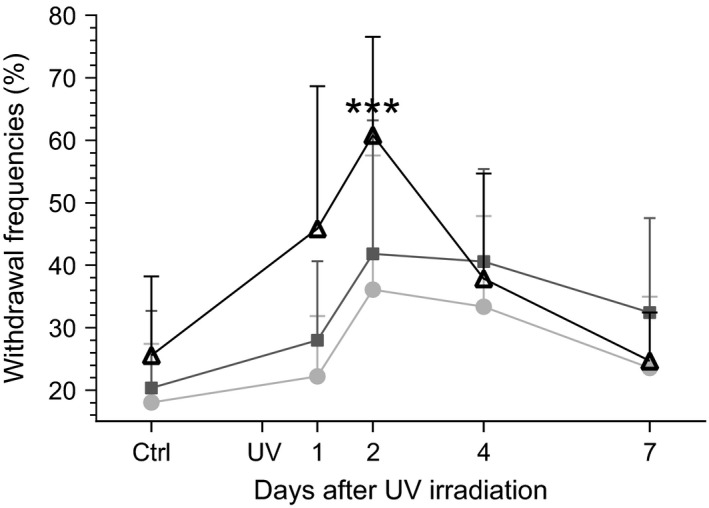
Time course of changes in frequency of withdrawal responses to CO_2_ laser stimulation following UVB irradiation of the hind paw skin. Mean and SEM of percentages of withdrawal responses upon CO_2_ laser stimulation within the Heel (open triangles), Pad (dark grey squares) and Digit (light grey circles) areas of the hind paw (cf. Fig. 1) are plotted; time scale after UVB irradiation is linear. The control (Ctrl) value before UVB irradiation (UV) represents a mean of 11 test occasions performed postsurgery (cf. Supporting Information Fig. S1). ***p < 0.001; one‐way anova followed by Bonferroni's multiple comparison test (entry f) in Supporting Information Table S1).

Like the cortical activity described above, thresholds of responses to mechanical stimuli (using dynamic plantar aesthesiometry) and latencies of responses to thermal stimuli (Hargreaves plantar test) changed after UVB irradiation as compared to the control condition, but with a conspicuously different time course (Fig. 4). In contrast to the distinct peak in cortical responses on Day 1, significantly lowered thresholds of responses to mechanical stimuli were observed on Day 4 (*p* < 0.001, entry d) in Supporting Information Table S1) only and significantly shortened latencies of responses to thermal stimuli were observed on Days 2 and 4 (*p* < 0.05, entry e). By Day 7 thresholds and latencies were similar to those before UVB irradiation. Notably, frequencies of limb withdrawals on CO_2_ laser stimulation of Heel peaked on Day 2 after UVB irradiation (*p* < 0.001, entry f) in Supporting Information Table S1), whereas no significant increases in frequency were seen for stimulation of Digits and Pad (Fig. 5).

### 3.3 Control experiments

To ascertain that UVB irradiation affected the skin locally as intended, we characterized the changes in blood flow that the irradiation gave rise to. Indeed, the irradiated skin showed a clear erythema on Day 1 after UVB irradiation, as indicated by increased blood flow: Mean blood flow before irradiation was 200 ± 31 perfusion units (PU) and 387 ± 38 PU on Day 1 (*p* < 0.05, *n* = 7 entry k) in Supporting Information Table S1). Animals did not develop blisters as a result of the irradiation and no spontaneous pain behaviour such as licking or flinching was observed (cf. some other inflammatory pain models).

In view of the focus on hyperalgesia in this study, it was important to establish that the electrode implant itself did not cause sensitization of nocifensive behaviour. We therefore examined long‐term alterations in withdrawal responses before and after surgery, but before UVB irradiation. As seen in Fig. S1, there was no significant difference between pre‐ and postsurgery with respect to withdrawal threshold to mechanical stimulation or withdrawal latencies upon thermal stimulation, indicating that the implantation of the multichannel electrode had no effects on nociceptive responses.

## 4 Discussion

We addressed the need for an animal model that could potentially provide important information on human perception of pain so as to complement current state‐of‐the‐art, commonly used models, which are typically based on behavioural measures of pain (Barrot, 2012; Dolgin, 2010; Woolf, 2010). Using a novel, biocompatible multichannel electrode that was implanted in S1 using novel in‐house techniques, combined with a well‐established translational method for inducing hyperalgesia and for which there is substantial documentation of altered response patterns in wide dynamic range neurons in the spinal cord (Lopes and McMahon, 2016; O'Neill et al., 2015; Sikandar et al., 2013), we present a model of pain in freely moving animals for electrophysiological studies conducted over a relatively long time course (weeks rather than days). Crucially, we demonstrate that, with respect to time course of both primary and secondary hyperalgesia, the altered nociceptive‐evoked activity in S1 in awake rats after UVB irradiation of the skin appears consistent with perceptual changes previously described in humans during analogous conditions (Bishop et al., 2009; Gustorff et al., 2013). In contrast, nociceptive withdrawal reflexes thresholds in the same animals, were significantly decreased only from the UVB irradiated skin area, confirming previously published data (Bishop et al., 2010; Davies et al., 2011; Hoffmann and Schmelz, 1999) and the time course of this primary hyperalgesia was significantly different compared to that found in S1 (and, hence, as compared to what is found in humans (Gustorff et al., 2013; Nir et al., 2008)). These findings indicate that there are important differences in the underlying encoding of nociceptive information during development of hyperalgesia in S1 and spinal sensorimotor circuits in conscious, freely moving animals.

There is strong evidence that implicates S1 in human pain perception. Indeed, it has been demonstrated that while many cortical areas are activated by heat stimulation of the skin, only the evoked potentials in S1 are strongly correlated with the perceived magnitude of pain in humans (Gustorff et al., 2013; Nir et al., 2008). Although these findings do not rule out the possibility that other cortical, or possibly even subcortical, areas are involved in the perception of pain magnitude (Nahman‐Averbuch et al., 2014; Verne et al., 2004), they do indicate that measurements of evoked potentials in S1 have the potential of being a highly useful electrophysiological biomarker for pain. Previous animal studies on the nociceptive input to S1 have been carried out in acute experiments, mainly under anaesthesia (Jensen et al., 2011; Kuo et al., 2009; Schouenborg et al., 1986; Zhang et al., 2011). To our knowledge, long‐term changes in S1 processing of nociceptive information, such as those occurring during development of UVB‐induced hyperalgesia, have not been studied previously in awake, freely moving animals. Our data obtained on day 1 after UVB irradiation confirm previously obtained results from animals under anaesthesia (Jensen et al., 2011), with respect to potentiation of both nociceptive input to S1 and lumbar wide dynamic range neurons (O'Neill et al., 2015; Sikandar et al., 2013) some of which are likely to have been ascending neurons. More importantly, however, the novel techniques used here provide the advantages of allowing us to follow the entire time course of events without the potentially confounding effects of anaesthesia. Notably, we found that the nociceptive input from both UVB irradiated and non‐irradiated skin areas to S1 followed a similar time course to that previously shown in psychophysical studies of primary and secondary hyperalgesia in human subjects (Gustorff et al., 2013). In humans, a UVB induced sunburn is accompanied by a well characterized development of primary (within the irradiated area) and secondary (outside the irradiated area) hyperalgesia, both with a peak 24–36 h after induction and then declining over the next 7 days (Bishop et al., 2009; Gustorff et al., 2004, 2013). Although secondary hyperalgesia to radiant heat stimulation has to our knowledge not been described in humans, a significant secondary hyperalgesia to pin prick has been reported after UVB irradiation (Gustorff et al., 2013). Notably, heat stimulation, if delivered to a small skin area (such as delivered with the CO_2_ laser stimulation used in this study), also gives rise to a pain sensation of pin prick in human skin (Nilsson and Schouenborg, 1999; Voegelin et al., 2003). It is thus conceivable that the potentiation of S1 potentials evoked from non‐irradiated skin is dependent on mechanisms analogous to those underlying secondary hyperalgesia in humans. Given the similarities with respect to time course of both primary and secondary hyperalgesia, it appears that our new animal model captures important features of human perception of pain magnitude.

In this study, we recorded both evoked potentials and multiunit discharges from deep S1 in awake, freely moving rats for several weeks. While both measures were significantly increased on day 1 they followed different time courses thereafter. The magnitude of the nociceptive‐evoked potentials declined slowly over the subsequent days back to baseline levels, whereas the evoked neural spike discharges was back to control levels, or as in the case of responses elicited from digits, significantly below control level already on day 2. Hence, there is no simple relationship between the magnitude of the evoked potentials and S1 neuronal discharges. Although cortical‐evoked potentials can be assumed to mainly reflect summation of synaptically evoked activity in cortical neurons caused by the incoming thalamocortical nociceptive input (Eggermont and Smith, 1995; Kulics and Cauller, 1986), the evoked neural discharges may reflect the corticofugal output, which is dependent not only on incoming spino‐thalamo‐cortical synaptic input but also on the excitability of cortical neurons and intrinsic cortical [or intra‐cortical] processing (Jiang et al., 1990; Medini, 2011). The reduction in spontaneous activity, prior to stimulation, observed on day 2 suggests a reduced excitability in the cortical nociceptive neurons studied during this time period. Given that the peak increase in reflex excitability occurring on day 2 (see also Davies et al., 2005; Bishop et al., 2010; O'Neill et al., 2015) may cause a reduced activation of the plantar skin mechanoreceptors, it may be speculated that a reduced cortical excitability to some extent is related to a reduction in background excitatory input from the hind paw. Alternatively, it is conceivable that the population of cortical neurons studied exerts a significant spinal control of reflex transmission.

In line with the observations of Bishop et al. (2010), withdrawal reflexes from the UVB‐irradiated skin area were significantly potentiated on day 2 for heat stimulation and on day 4 for mechanical stimulation (Bishop et al., 2007). The time courses of decreases in withdrawal reflex latency found here, using conventional radiant heat and mechanical stimulation (Hargreaves et al., 1988; Lambert et al., 2009) are consistent with those previously reported in awake rats after UVB irradiation of the skin (Bishop et al., 2007; Saadé et al., 2008; O'Neill et al., 2015; Lopes and McMahon, 2016) but significantly delayed compared to the potentiated CO_2_ laser evoked nociceptive potentials and neuronal responses in S1 as revealed here by recordings in a subgroup of the same animals. Our data on frequency of CO_2_ laser‐evoked reflexes also confirm the lack of a significant reflex potentiation from non‐irradiated skin, as previously reported (Bishop et al., 2010; Davies et al., 2005, 2011). The discrepancies in THIS study between spinal sensorimotor and cortical circuits found in the same animal with respect to topographical and temporal characteristics of primary and secondary hyperalgesia thus indicate important functional differences between nociceptive processing in nociceptive motor and nociceptive sensory systems. While little is known about the processing of nociceptive information in cortical circuits, the functional organization of the nociceptive withdrawal reflex system has been characterized in some detail (Sandrini et al., 2005; Schouenborg, 2008). In short, this reflex system is organized as a modular system, where each reflex module is primarily concerned with the control of a single muscle and encodes (weighs) sensory input from the skin surface area withdrawn by the muscle upon contraction. For each of these modules, the encoding of nociceptive input from respective receptive field is task specific. The same sensory encoding principles apply to cerebellar modules concerned with coordination and adaptation of limb movements (Apps and Garwicz, 2005). By analogy, it may be speculated that S1 processing is also task specific, but concerned with more sensory‐related functions such as an analysis of the impact of the injury on the integrity of the body.

### 4.1 Concluding remarks

From previous studies in anaesthetized rats, it is known that the magnitude of the nociceptive‐evoked potentials in S1 is enhanced following sensitization of the ascending spinal nociceptive pathways and reduced by analgesics administered topically on the spinal cord (Kalliomäki et al., 1998) or systemically (Granmo et al., 2013), i.e. manipulations that also result in corresponding changes in pain magnitude in humans (Oertel et al., 2008). The present findings in the awake rat, that long‐term S1 recordings capture important topographical and temporal features of human perception of UVB‐induced primary and secondary hyperalgesia indicate that such recordings have the potential of providing a powerful translational biomarker for hyperalgesia. It should be kept in mind, however, that human pain is multidimensional and comprises sensori – discriminative, affective and motor aspects (Bonica, 1979). An ideal animal model of pain should thus incorporate all these aspects. Hence, it may be argued that a combination of behavioural and perceptually related assessments is advantageous when, for example evaluating the properties of putative analgesic treatments.

## Author contributions

All authors substantial contributions to conception and design, or acquisition of data, or analysis and interpretation of data. All authors discussed the results, commented on the manuscript and gave final approval of the version to be published.

## Supporting information


**Figure S1** Controlling for potential effects of surgery and implantation on mechanical withdrawal threshold (upper panel) and heat withdrawal latency (lower panel).

**Table S1** Statistical tests used.

**Appendix S1** Methods.
