# Supplementary material for: Discrepancies between cortical and behavioural long‐term readouts of hyperalgesia in awake freely moving rats
Source: Eur J Pain. 2016 May 5;20(10):1689–99. doi: 10.1002/ejp.892 (PMC5096034; doi:10.1002/ejp.892)
Supplement: Supplementary file 1 — Figure S1 Controlling for potential effects of surgery and implantation on mechanical withdrawal threshold (upper panel) and heat withdrawal latency (lower panel). [file EJP-20-1689-s001.pdf]

FIG S1

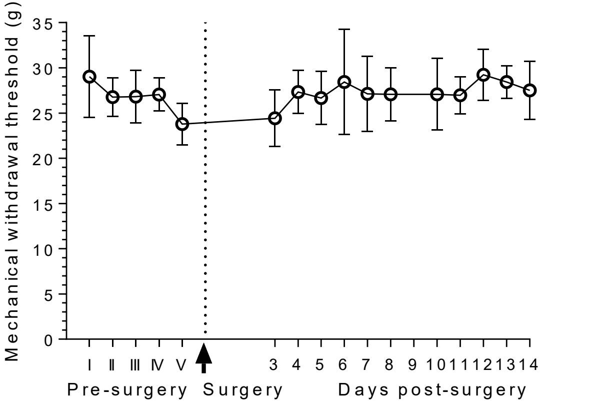

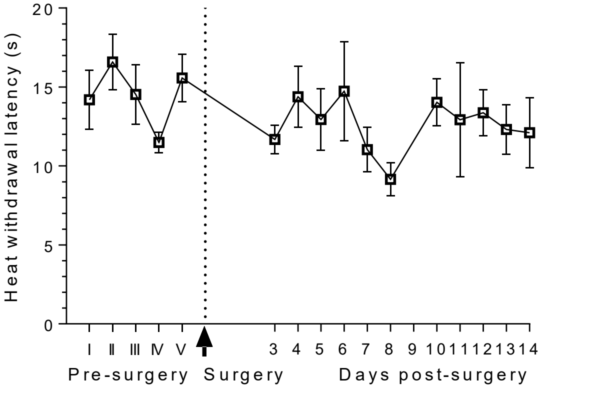

FigureS1: Controlling for potential effects of surgery and implantation on mechanical withdrawal threshold (upper panel) and heat withdrawal latency (lower panel). Roman numerals indicate consecutive test occasions Pre-surgery. Arrows indicate time of surgery. Time course post-surgery is indicated by daily tests (except Day9); symbols depict mean and SEM. No significant changes were seen due to surgery (one-way ANOVA (entries g) resp. h) in tableS1) followed by a Tukey's post hoc test; n=7 for both groups). Animals were UVB irradiated (cf. Figs 4 and 5) after testing on Day14.
