# Supplementary material for: Discrepancies between cortical and behavioural long‐term readouts of hyperalgesia in awake freely moving rats
Source: Eur J Pain. 2016 May 5;20(10):1689–99. doi: 10.1002/ejp.892 (PMC5096034; doi:10.1002/ejp.892)
Supplement: Supplementary file 2 — Table S1 Statistical tests used. [file EJP-20-1689-s002.docx]

**TableS1**

| No | Name | Data Structure | Type of test | Power |
| --- | --- | --- | --- | --- |
| a) | ad and c units spike response, hind paw laser stim., post UV compared to baseline | log-normal | 1-tailed t-test, Bonferroni correction n=4 | Laser response: p<0.001 (all areas day 1)  p<0.05 (pad day 4),  p>0.05 other days. |
| b) | evoked potentials, hind paw laser stim., post UV compared to baseline | non-normal distribution | Wilcoxon rank sum test, Bonferroni correction n=4 | p<0.01  (digits day 1),  p<0.001 (pad heel day 1, all areas day 2, heel day 4) |
| c) | ad- and c-unit identification vs baseline | log-normal | 2 sided t-test | check if p<0.05 for identification of unit |
| d) | Mean and SEM of withdrawal thresholds to mechanical stimulation post UV | normal | one-way ANOVA, | p<0.001 (day 4) |
| e) | withdrawal latencies to heat stimulation post UV | normal | one-way ANOVA | p<0.05 (day 2 and 4) |
| f) | withdrawal frequencies to laser stimulation | binomial distribution (normal distribution assumed since n is large) | one-way ANOVA | p< 0.001 |
| g) | mechanical withdrawal threshold post operation | normal | one-way ANOVA | non-significant |
| h) | heat withdrawal latency post operation | binomial distribution (normal distribution assumed since n is large) | one-way ANOVA | non-significant |
| i) | ad and c units spike response, hind paw laser stim., post UV compared to baseline | log-normal | 2-tailed t-test, Bonferroni correction n=4 | Less than baseline day 2: p<0.05 (digits only) |
| j) | ad and c units baseline activity, post UV compared to baseline | log-normal | 2-tailed t-test, Bonferroni correction n=4 | baseline activity day 1 post UV increased for heel area: p< 0.05 |
