# Supplementary material for: Discrepancies between cortical and behavioural long‐term readouts of hyperalgesia in awake freely moving rats
Source: Eur J Pain. 2016 May 5;20(10):1689–99. doi: 10.1002/ejp.892 (PMC5096034; doi:10.1002/ejp.892)
Supplement: Supplementary file 3 — Appendix S1 Methods. [file EJP-20-1689-s003.docx]

# Supporting information

**Methods**

***Ethical approval and animals used***

These regulations, including directives from the European Union, follow the law on animal welfare legislated by the Swedish parliament. The animals were kept in the animal facilities of the Biomedical Center at Lund University and experiments were carried out at the Section for Neurophysiology. All animals received food and water *ad libitum* and were kept in a 12-hour day–night cycle at a constant environmental temperature of 21°C and 65% humidity. We used female rats to ensure that the size of the animals was stable during the approximately 1 month long test period. While the estrous cycle has been shown to affect nociceptive thresholds, this appears to be regionally specific (Yamashita et al. 2015) and does not include the skin areas tested in the present study. Also, the experiments were not synchronized with the estrous cycle, minimizing the risk of any systematic effects on nociceptive thresholds.

***UVB irradiation***

UVB irradiation produces a skin inflammation and a dose-dependent hyperalgesic state (Bishop et al., 2007). Here, rats were anaesthetized with isoflurane (1.3–2.0%) in a mixture of 40% oxygen and 60% nitrous oxide. Half of the hind paw was covered with a UV-blocking film from an FR-4 clad board (ELFA, Sweden) and tin foil covered with paper protected remaining parts of the body from UV exposure. Seven animals were irradiated with 1.2 Jcm^-2^ (Fig. 1), using a Philips UVB TL/01 narrowband lamp (PL-S 9W/01, λ = 300–320 nm). This intensity has been reported to be just below the threshold for blistering(Bishop et al., 2007). Before every exposure, the lamp was left on for 3 min to allow the UVB intensity to stabilize. UVB intensity was measured before every exposure using a Varicontrol UV/PDT meter and skin tester (Herbert Waldmann GmbH & Co. KG, Germany). Recordings of evoked potentials commenced 20–24 h after irradiation. At this time, discrete to moderate redness of the irradiated skin, but no skin lesions or scarring, was seen on the irradiated skin in each rat, confirming the presence of an inflammatory process (Bishop et al., 2009).

### ***Assessment of mechanical thresholds for withdrawal using dynamic plantar aesthesiometer***

Withdrawal responses to mechanical stimuli were tested from the proximal part of the right hind paw (Fig. 1) using a dynamic plantar aesthesiometer (Ugo Basile, Italy). The stimulus was applied via an actuator filament (O.D 0.5 mm). A computer controlled linear force ramp of 0.4 g/s was applied, which stopped the measurement upon paw withdrawal. The withdrawal threshold was calculated as the average of four consecutive tests, with a minimum of 1 minute between stimulations. A cut-off of 50 g was set to prevent tissue damage.

### ***Assessment of thermal thresholds for withdrawal using Hargreaves plantar test***

The degree of thermal nociception was determined by measuring paw withdrawal latency to radiant thermal stimulation. The withdrawal latency tests were performed three times with ≥ 5 minutes interstimulus interval. A cut-off of 30 s was imposed to prevent tissue damage. If responses were not evoked from three consecutive tests, five tests were performed and the average of three out of five tests was used. The set-up consisted of a clear cubicle (17 x 22 x 14 cm) on top of a glass floor (Hargreaves acrylic cubicle, Plantar test cat. 7370). A radiant light source (Halogen, Mod. 64607 Osram 8V, 50W) mounted on a movable holder below the floor was positioned under the heel of the right hind paw to deliver a thermal stimulus (Fig. 1). A photocell detected interruption of the light beam reflection upon withdrawal of the paw, where after the I.R. generator and timer were automatically switched off, determining the withdrawal latency.

***Nociceptive stimulation***

A CO_2_ laser (Irradia, Sweden; model 315M Superpulse, wavelength 10.6 μm, beam diameter 3.0 mm) with a 10 W output power was used to elicit nociceptive neural activity and C fibre evoked potentials.

***Multichannel electrode***

A microwire array electrode was built in house. The electrode consisted of 29, 12 µm platina-irridium wires insulated with paryleneC (Paratech, Sweden) and embedded in gelatine type A (2%; Sigma-Aldrich Co, USA) for optimal stiffness during insertion into the cortex (Lind et al., 2010). One of the electrodes was exposed for a length of 0.2 mm at the tip and used as local reference. The wires and a 150 µm thick platinum ground lead were soldered to an electronic chip and the connections were covered with Epotek GE116-1 (bisfenol F epoxiharts, Epoxy technology, USA).

***Surgery and implantation of multichannel electrodes***

The head was shaved and then the rat was mounted on a stereotactic frame (KOPF Instruments, USA) under a stereomicroscope (Leica Microsystems, M651, Germany). The surgical area of the scalp was disinfected using 70% ethanol and a 3 cm midline incision was made to expose the skull. Thereafter, the skin was retracted and the skull cleaned from tissue. A hole (O.D 0.9 mm) was drilled approximately 2 mm anterior of bregma and 1 mm lateral to the midline in which a stainless screw was mounted and attached to the ground electrode with silver conductive paint (Electrolube®, HK Wentworth Ltd, UK). Two additional screws were mounted caudal of lambda for anchoring the implant. The stereotactic coordinates for the S1 hind paw area were defined as 1 mm caudal of bregma and 2.4 mm lateral to the midline and a rectangular (3 x 2 mm) craniotomy was made (Jensen et al., 2011; Kalliomäki et al., 1993). The exposed dura mater was removed and the surface was covered with artificial cerebrospinal fluid. The electrodes were mounted in a hydraulic micromanipulator (KOPF Instruments, USA) and implanted at a speed of 10 µm/s to the depth of 1.6 mm in the left cerebral hemisphere. In order for the gelatine to start dissolving, and thereby to allow unfolding of the micro wires on further insertion, a 5 minute pause was made at 0.5 mm depth. Dental cement (GC FujiCEM Automix) was used to cover the hole in the skull and to embed the wire bundle, leaving the electrode contact free.

After releasing the animal from the stereotactic frame it was given 10.5 mg/kg s.c. injection Antisedan vet (atipamezole hydrochloride; Orion pharma, Finland). This injection served as an antidote to the anaesthesia. For postoperative analgesia, the rats were injected s.c. 1 mg/kg Temgesic (buprenorfin; Schering-Plough, Belgi­um). In cases when the surgery lasted longer than one hour, 5 ml saline was injected i.p.

***Data recording and analysis***

LFPs were sampled at 1017 Hz and filtered using a low-pass (<300 Hz) FIR filter. Also, 50 Hz noise was removed from the signals using a digital notch filter. In order to remove low-frequency baseline offset, the mean of the trial signal from 500 to 50 ms before stimulus was subtracted from each trial. Trials were rejected if they contained noise in their prestimulus baseline corresponding to more than 3 SD, with SD estimated using all trials of a recording session. 0.3 % of the trials were removed based on this criterion.

The onset of each CO_2_ laser evoked response was defined as the maximum of the smoothened second derivative (using a moving average filter with window length of 0.05 s) of the smoothened means in a range from 190 to 350 ms, corresponding to an increased steepness of the “uphill” curve. The amplitude at the onset time was defined as the baseline for the evoked potential. The end of the response was defined as when the evoked potential crossed the baseline amplitude.

***Reference list***

Bishop, T., Ballard, A., Holmes, H., Young, A.R., McMahon, S.B. (2009). Ultraviolet-B induced inflammation of human skin: characterisation and comparison with traditional models of hyperalgesia. *Eur J Pain* 13, 524–532.

Bishop, T., Hewson, D.W., Yip, P.K., Fahey, M.S., Dawbarn, D., Young, A.R., McMahon, S.B. (2007). Characterisation of ultraviolet-B-induced inflammation as a model of hyperalgesia in the rat. *Pain* 131, 70–82.

Jensen, T., Granmo, M., Schouenborg, J. (2011). Altered nociceptive C fibre input to primary somatosensory cortex in an animal model of hyperalgesia. *Eur J Pain* 15, 368–375.

Kalliomäki, J., Weng, H.R., Nilsson, H.J., Schouenborg, J. (1993). Nociceptive C fibre input to the primary somatosensory cortex (SI). A field potential study in the rat. *Brain Res* 622, 262–270.

Lind, G., Linsmeier, C.E., Thelin, J., Schouenborg, J. (2010). Gelatine-embedded electrodes--a novel biocompatible vehicle allowing implantation of highly flexible microelectrodes. *J Neural Eng* 7, 046005.

Yamashita, H., Zeredo, J.L., Kimoto, M., Nihei, Z., Asahina, I., Kaida, K., Toda, K. Body site-dependent changes in pain threshold during the estrous cycle in rats. Psychology & Neuroscience, Vol 8(3), Sep 2015, 414-422
